# Interim-treatment quantitative PET parameters predict progression and death among patients with hodgkin's disease

**DOI:** 10.1186/1748-717X-7-5

**Published:** 2012-01-19

**Authors:** Diane Tseng, Leelanand P Rachakonda, Zheng Su, Ranjana Advani, Sandra Horning, Richard T Hoppe, Andrew Quon, Edward E Graves, Billy W Loo, Phuoc T Tran

**Affiliations:** 1 Department of Radiation Oncology, Stanford University School of Medicine, 875 Blake Wilbur Dr., Stanford, CA 94305 USA; 2 Department of Health Research and Policy, Biostatistics, Stanford University School of Medicine, 300 Pasteur Dr., Stanford, CA 94305 USA; 3 Department of Medicine, Medical Oncology, Stanford University School of Medicine, 875 Blake Wilbur Dr., Stanford, CA, 94305 USA; 4 Department of Radiology, Stanford University School of Medicine, 300 Pasteur Dr., Stanford, CA, 94305 USA; 5 Department of Radiation Oncology and Molecular Radiation Sciences, Sidney Kimmel Comprehensive Cancer Center, Johns Hopkins Medical Institutes, 1550 Orleans St., CRB2, Baltimore, MD 21231 USA; 6 Department of Oncology, Sidney Kimmel Comprehensive Cancer Center, Johns Hopkins Medical Institutes, 1550 Orleans St., CRB2, Baltimore, MD 21231 USA

**Keywords:** Hodgkin's disease, PET, metabolic tumor volume, quantitative PET parameters, survival

## Abstract

**Purpose:**

We hypothesized that quantitative PET parameters may have predictive value beyond that of traditional clinical factors such as the International Prognostic Score (IPS) among Hodgkin's disease (HD) patients.

**Methods:**

Thirty HD patients treated at presentation or relapse had staging and interim-treatment PET-CT scans. The majority of patients (53%) had stage III-IV disease and 67% had IPS ≥ 2. Interim-treatment scans were performed at a median of 55 days from the staging PET-CT. Chemotherapy regimens used: Stanford V (67%), ABVD (17%), VAMP (10%), or BEACOPP (7%). Hypermetabolic tumor regions were segmented semiautomatically and the metabolic tumor volume (MTV), mean standardized uptake value (SUVmean), maximum SUV (SUVmax) and integrated SUV (iSUV) were recorded. We analyzed whether IPS, absolute value PET parameters or the calculated ratio of interim- to pre-treatment PET parameters were associated with progression free survival (PFS) or overall survival (OS).

**Results:**

Median follow-up of the study group was 50 months. Six of the 30 patients progressed clinically. Absolute value PET parameters from pre-treatment scans were not significant. Absolute value SUVmax from interim-treatment scans was associated with OS as determined by univariate analysis (p < 0.01). All four calculated PET parameters (interim/pre-treatment values) were associated with OS: MTV_int/pre _(*p *< 0.01), SUVmean_int/pre _(*p *< 0.05), SUVmax_int/pre _(*p *= 0.01), and iSUV_int/pre _(*p *< 0.01). Absolute value SUVmax from interim-treatment scans was associated with PFS (p = 0.01). Three calculated PET parameters (int/pre-treatment values) were associated with PFS: MTV_int/pre _(*p *= 0.01), SUVmax_int/pre _(*p *= 0.02) and iSUV_int/pre _(*p *= 0.01). IPS was associated with PFS (*p *< 0.05) and OS (*p *< 0.01).

**Conclusions:**

Calculated PET metrics may provide predictive information beyond that of traditional clinical factors and may identify patients at high risk of treatment failure early for treatment intensification.

## Background

Positron emission tomography [1] imaging using [^18^F]fluorodeoxyglucose serves as a valuable functional imaging modality in patients with lymphoma [2-4]. The ability of PET to distinguish between viable tumor and necrosis or fibrosis in residual masses provides an advantage over conventional imaging using computed tomography [5] or magnetic resonance imaging [6-8]. The high sensitivity and specificity of [^18^F]FDG-PET imaging for lymphoma staging has been demonstrated in previous studies [9-12]. Fused FDG-PET and CT imaging combines functional with anatomic information about the tumor and is now routinely used in radiation treatment planning. PET-CT is now strongly recommended by the International Harmonization Project in Lymphoma for staging and reassessment of FDG-avid, potentially curable lymphomas such as Hodgkin's disease and diffuse large B-cell lymphoma [13].

In Hodgkin's disease, long-term disease control is high and late treatment toxicity and secondary cancers are emerging as new challenges. It is therefore increasingly important to develop individualized risk-adapted treatment approaches for this disease. Several recent studies have demonstrated the role of [^18^F]FDG-PET in predicting clinical outcome for patients with Hodgkin's disease. Advani et al showed that a positive [^18^F]FDG-PET scan following completion of Stanford V chemotherapy was predictive of freedom from progression, even after controlling for bulky disease and International Prognostic Score (IPS) greater than 2 [14]. The potential clinical utility of [^18^F]FDG-PET scan may be extended even further. Although the optimal timing for evaluating early response to treatment is yet to be defined, a recent joint Italian-Danish study has prospectively shown that [^18^F]FDG-PET scan following two cycles of AVBD chemotherapy is superior than IPS in predicting progression-free survival in patients with Hodgkin's disease [4].

Evaluation criteria of [^18^F]FDG-PET scans were qualitative in these studies. Variability in interpretation of PET-CT scans, particularly in cases with faint residual uptake or "intermediate-positive" scans, limits the broad clinical utility of this tool. Whether quantitative PET parameters is a predictive factor for disease progression in Hodgkin's disease is not well established. Metabolic tumor volume (MTV) has been described in a previous study incorporating patients with Hodgkin's disease and non-Hodgkin's lymphoma to be an independent prognostic factor, but this study had a short follow-up period of 12.7 months and it did not examine the prognostic value of interim-treatment PET parameters [15]. We hypothesize that pre- and interim-treatment quantitative PET parameters may provide increased predictive strength beyond traditional clinical factors such as the International Prognostic Score (IPS).

## Methods

### Patients

We conducted an IRB approved retrospective review of the medical records of patients who underwent [^18^F]FDG-PET scanning at Stanford Hospital and Clinics between January 2003 and June 2005 in order to maximize the length of clinical follow-up. During this study period, 3, 548 [^18^F]FDG-PET scans were performed. Of the patients scanned, we identified 57 adult and pediatric patients who were evaluated for Hodgkin's disease at presentation or relapse and had interim-treatment PET-CT scans (Table 1). It was the general policy to obtain an interim PET-CT scan, but of 57 patients, we identified 30 patients with corresponding initial staging PET-CT scans performed at our institution that had subsequent clinical follow-up. Patients received chemotherapy with Stanford V [16], ABVD (1), VAMP [17], or BEACOPP [18]. Choice of chemotherapy and duration of treatment varied for patients with early and advanced disease. Following completion of chemotherapy, radiation therapy (for a total of 20 Gy, 25.5 Gy, or 36 Gy) was administered to involved sites according to institutional protocols. Among the patients in this study, one required treatment intensification to ifosfamide, carboplatin, and etoposide (ICE) chemotherapy interim-treatment. One patient received treatment modification due to poor venous access and switched from ABVD to CH1VPP (chlorambucil, vinblastine, procarbazine and prednisolone) after two cycles. Another patient received the last 2 cycles of ABVD chemotherapy without bleomycin due to pneumonitis. Date of diagnosis, staging and interim-treatment PET scans, date of local recurrence, date of distant progression, date of last follow-up or death, and disease status were recorded.

**Table 1:** Patient Characteristics

Parameter		No. of patients (%)
Gender	Male	20 (67)
	Female	10 (33)
Age	Range	9 - 75
	Median	19.5
	> = 45 yo	3
Stage	I A	1 (3)
	I B	0
	II A	10 (33)
	II B	3 (10)
	III A	5 (17)
	III B	4 (13)
	IV A	1 (3)
	IV B	6 (20)
Bulky Disease		12 (40)
Extranodal Disease		15 (50)
IPS	0 - 2	21 (70)
	> = 3	9 (30)

### PET Imaging protocol

PET images were acquired on a GE Discovery LS PET-CT scanner (GE Medical Systems, Milwaukee, WI) as previously described [19]. In brief, each patient received 12 to 18 mCi of FDG 45 to 60 min before PET/CT imaging. PET data were acquired using about seven bed positions, with 3 to 5 min of acquisition time per position. Patients were injected between 12-18 miCi of FDG based on patient weight. The amount of FDG injected for individual patients did not vary significantly provided that their weights did not change significantly between the scans.

### Measurement of quantitative PET parameters

**Figure 1:**
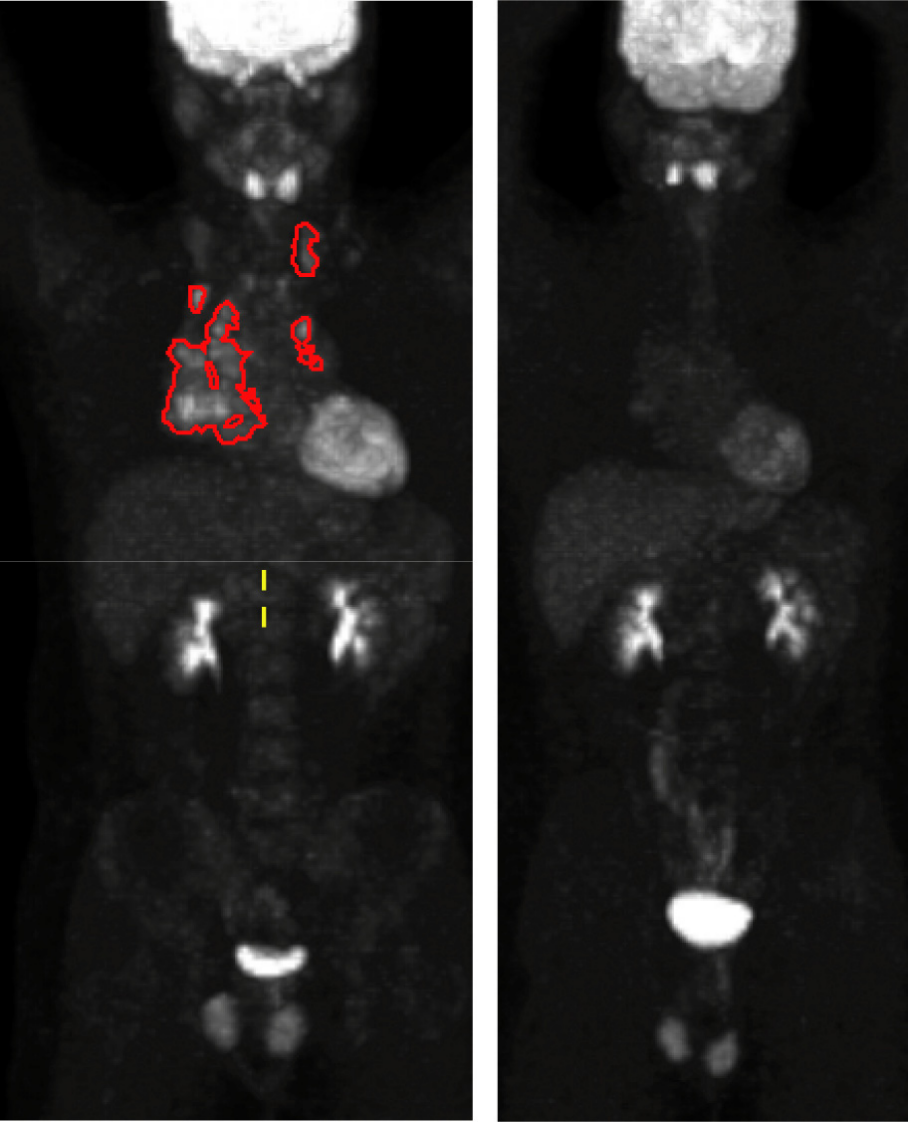
**Maximum intensity projection view of ^18^F-fluorodeoxyglucose-positron emission tomography image with overlay of segmented metabolic tumor volumes (MTV)**. This is a representative pre-treatment PET image (left; MTV = 94 mL, SUVmax = 8.9, SUVmean = 3.4, and iSUV = 319.8) and interim-treatment PET image (right; absolute value and calculated MTV = 0 mL, SUVmax = 0, SUVmean = 0, and iSUV = 0).

Hypermetabolic tumor foci were segmented with a software application, RT_Image, as previously described (see Figure 1 for illustrative example) [19]. In brief, images were displayed as a maximum intensity projection (MIP) and then each metabolically active lesion was selected semi-automatically with reference to the patient's radiology report. Next, RT_Image was used to determine metabolic tumor volume (MTV), the total volume of all selected tumors in milliliters, mean standardized uptake value (SUV_mean_), maximum SUV (SUV_max_) and integrated SUV (iSUV), defined as a product of MTV and SUV_mean _[19]. We herein refer to these values as absolute value PET parameters.

Interim-treatment scans were performed at a median of 55 days from the staging PET-CT, corresponding to 2 cycles of Stanford V chemotherapy which was the most commonly used regimen in this cohort of patients. Interim-treatment scans were then registered to the corresponding pre-treatment scans and the identical regions interrogated. The ratio of interim- to pre-treatment PET parameters was calculated, for instance, as follows: interim-treatment SUV_max_/pre-treatment SUV_max_. We herein refer to these values as calculated PET parameters.

### Statistical Analysis

Clinical relapse was defined as death, biopsy-proven recurrence or radiographic findings leading to a change in management based on clinical judgment. The Cox proportional hazards model was used for univariate analysis to assess the effect of patient variables and treatment factors on the end points described above. Survival graphs were generated by the product limit method of Kaplan and Meier and log-rank analysis was utilized for differences between proportions. Analysis was facilitated using R freeware by the GNU project(5) and Prism v4.0 by GraphPad [20].

## Results

### Patient characteristics

Of the 30 patients in this cohort, the majority (53%) had stage III-IV disease, 67% had an IPS of 2 or greater, and 30% had an IPS of 3 or greater. Patient characteristics, including patient age, gender, stage, bulky disease, extranodal disease, IPS score, and chemotherapy received, are summarized in Table 1.

### Outcomes

**Figure 2:**
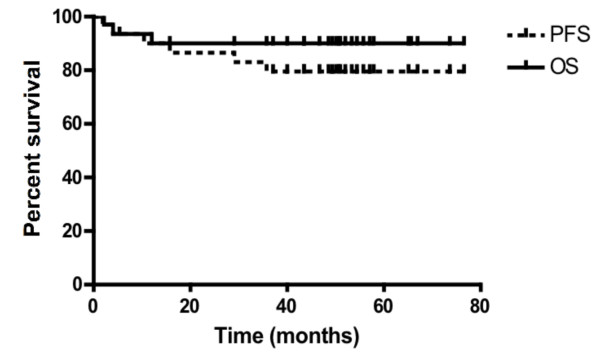
**Progression-free and overall survival**. Progression free survival (PFS, solid line) and overall survival (OS, dashed line) are represented. Four-year PFS was 80% and OS was 90%.

At the time of this analysis, median follow-up of the study group was 50 months. Six of the 30 patients progressed clinically (Table 2). We did not observe any non-cancer deaths (for instance, deaths unrelated to Hodgkin's disease or its treatment) as the first event. The Kaplan-Meier 4-year progression-free survival (PFS) and overall survival (OS) for the entire cohort was 80% and 90%, respectively (Figure 2). Follow-up for mortality was complete for all 30 patients, with 4 dead and 26 alive at the time of analysis. The Kaplan-Meier median OS has not been reached.

**Table 2:** Characteristics of patients who have relapsed

Age	Sex	Stage	mid/pre tx MTV	mid/pre tx iSUV	Chemo	Clinical outcome	Survival
17	M	III A	0	0	Stanford V	Progressed at completion of chemo and RT	Alive
12	F	IV B	6	7	Stanford V	Progressed at completion of chemo and RT	Dead
75	M	IV B	141	172	ABVD	Dyspnea & fluid overload after cycle 4 of ABVD, h/o orthotopic heart transplant	Dead
40	M	III A	74	33	ABVD	Expanding large axillary mass, chemo suspended for surgical excision	Dead
56	M	IV B	7	9	BEACOPP	Died unexpectedly after 2 cycles chemo	Dead
26	M	III A	0	0	ABVD	Evidence of disease recurrence on PET-CT but asymptomatic	Alive

### PET metrics

The average MTV for the pre-treatment PET-CT scans was 344 mL (range 8 - 1496 mL). The average value for maximum SUV in pre-treatment PET-CT scans was 15.9 (range 5 - 44), for mean SUV 5.3 (range 3 - 10), and for iSUV 1841.6 (range 24 - 8907). The average MTV for the interim-treatment PET-CT scans was 44 mL (range 0 - 515 mL). The average value for maximum SUV in interim-treatment PET-CT scans was 4.0 (range 0 - 19.3), for mean SUV 2.1 (range 0 - 7), and for iSUV 223.5 (range 0 - 2983).

### Predictive value

**Figure 3:**
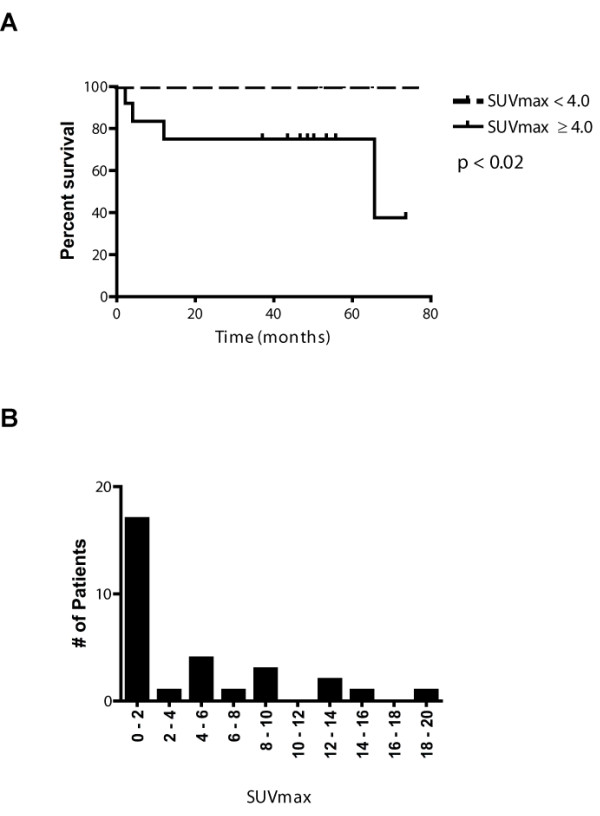
**(A) Overall survival (OS) represented by absolute value interim-treatment SUVmax with cutoff at average SUVmax = 4.0**. (B) Distribution of absolute value interim-treatment SUVmax values in this study.

PET parameters from pre-treatment scans did not significantly correlate with outcomes (results are summarized in Table 3). Absolute value SUVmax from interim-treatment scans was significantly associated with OS as determined by univariate Cox proportional hazards (p < 0.01). Absolute value MTV (p < 0.06), SUVmean (p = 0.07), and iSUV (p = 0.10) from interim-treatment scans did not reach significance for predicting OS in this analysis. All four calculated PET parameters (int/pre-treatment values) were associated with OS: MTV_mid/pre _(*p *< 0.01), SUVmean_mid/pre _(*p *< 0.05), SUVmax_mid/pre _(*p *= 0.01), and iSUV_mid/pre _(*p *< 0.01). Absolute value SUVmax from interim-treatment scans was significantly associated with PFS (*p *= 0.01). Three calculated PET parameters (int/pre-treatment values) were significantly associated with PFS: MTV_mid/pre _(*p *= 0.01), SUVmax_mid/pre _(*p *= 0.02) and iSUV_mid/pre _(*p *= 0.01). For illustrative purposes, OS (Figure 3A) is plotted with Kaplan-Meier analysis for SUVmax using the average SUVmax of 4.0 as the cutoff (see "Conclusion" for further discussion). The distribution of interim-treatment SUVmax values are displayed in Figure 3B. IPS was associated with PFS (*p *< 0.05) and OS (*p *< 0.01).

**Table 3:** Summary of Cox proportional hazards analysis of quantitative PET parameters

Parameter	PFS *p*-value	OS *p*-value
Pre-treatment PET Metrics		
SUV max	NS	NS
SUV mean	NS	NS
MTV	NS	NS
Interim PET Metrics		
SUVmax	0.01	< 0.01
MTV	NS	NS
iSUV	NS	NS
SUV mean	NS	NS
Calculated PET Metrics		
MTV_int/pre_	0.01	< 0.01
SUVmean_int/pre_	NS	< 0.05
SUVmax_int/pre_	0.02	0.01
iSUV_int/pre_	0.01	< 0.01

NS - not significant.

## Discussion

In our study, we have demonstrated that the absolute value SUV_max _from interim-treatment scans were significantly associated with OS and PFS on univariate analysis. In addition, the calculated PET parameters from interim-treatment/pre-treatment PET-CT scans, MTV_mid/pre_, SUVmax_mid/pre_, and iSUV_mid/pre_, were predictive for PFS and OS. Calculated SUV_mean _from interim-treatment/pre-treatment PET-CT scans is also predictive of OS. This is consistent with the hypothesis that the chemosensitivity of the tumor as measured by PET-CT early during treatment is predictive of clinical outcome.

These data suggest that absolute value and calculated PET metrics taken from quantitative analysis of PET imaging may augment the IPS for predicting PFS or OS. IPS is utilized for prognostication of patients with advanced Hodgkin's disease and not early stage disease. The development of prognostic and predictive tools for patients beyond standard clinical staging could be an important advancement for adaptive treatment approaches. Relapses in this study occurred only in patients with advanced stage disease, so we could not determine if quantitative PET parameters were predictive for early stage disease. For the patients in this study, PFS and OS are 100% for early stage, non-bulky disease (n = 9). In a larger group of 101 patients with favorable early stage disease treated with Stanford V chemotherapy followed by radiation therapy (20 or 30 Gy) according to established protocols at Stanford, FFP was 94% and OS was 97% [21]. In our study, four-year PFS for advanced disease (stage III and IV or early stage bulky disease) is 70% and OS is 85% (n = 21). In a larger group of 142 patients with advanced disease treated with Stanford V chemotherapy followed by 36 Gy to involved sites at Stanford, 5-year PFS was 89% and OS was 96%[16]. The small sample size in our study limits our ability to directly compare clinical outcomes between the patients in our study and the larger group of patients with Hodgkin's disease treated at Stanford.

We showed that absolute value interim-treatment SUV_max _was predictive for OS using the average SUV_max _of 4.0 as the threshold value. This threshold level was chosen for illustrative purposes to demonstrate that differences in clinical outcomes can be separated based upon quantitative PET metrics. This analysis points to the potential utility of a quantitative approach for cases that may be difficult to assess following chemotherapy. However, the optimal cut-off values to be used in Hodgkin's disease still needs to be further evaluated in prospective clinical studies. The optimal technique and threshold values for segmenting hypermetabolic tumor foci also warrants further examination.

We describe several quantitative PET parameters that may be potentially applicable for predicting clinical outcome in patients with Hodgkin's disease. In our study, PET parameters from pre-treatment scans were not significant. This may be due to the small sample size of our study. Alternatively, it may be that the chemosensitivity of the tumor is more important for predicting clinical outcome than the magnitude of metabolically active tumor burden at diagnosis. It is interesting to note that both interim-treatment absolute value and calculated (interim-treatment/pre-treatment) SUVmax were predictive for overall survival and progression free survival. We hypothesize that tumor chemosensitivity may be reflected in interim-treatment PET scan parameters. Absolute value interim-treatment MTV has the advantage of being more directly measured than calculated MTV, although calculated MTV has the advantage of reflecting the change in metabolic activity from baseline. The absolute value PET parameters were used to determine the calculated PET parameters (for instance, absolute value interim-treatment SUVmax/pre-treatment SUVmax). In our dataset, absolute value SUVmax and calculated SUVmax were highly correlated (Spearman R = 0.94 and p = 0.0001). It is also notable that 16 patients had interim-treatment SUVmax of 0, and as a result, calculated SUVmax also shared the same value 0 for 16 of the 30 patients. The best approach for applying quantitative PET data in the treatment of patients with Hodgkin's disease warrants further study in a prospective manner.

Our results although preliminary, are consistent with the joint Italian-Danish study showing that [^18^F]FDG-PET scan following two cycles of AVBD chemotherapy predicts progression-free survival in Hodgkin's disease patients. The interpretation of the PET-CT scans differs in our study in that we employ quantitative analysis rather than qualitative assessment. The establishment of quantitative methods of assessing PET-CT scans may aid in the interpretation of scans with minimal residual uptake or with "intermediate-positive" disease. It also has the potential for standardizing the interpretation of scans to reduce the variability in technique among clinical trials and across different institutions. Since a PET-CT is generally performed to assess the extent of disease before and during treatment for patients with Hodgkin's disease, quantitative analysis would be a practical and cost-effective strategy for incorporating these data into clinical practice.

The limitations of our study are the relatively small number of patients, relatively small number of clinical events (progression and/or death), and retrospective method. Due to the small number of patients, we were not able to select a more homogenous population of patients receiving identical chemotherapy regimen, e.g. our stage I-II patients. Due to the low numbers of events we were also unable to perform a multivariate analysis to exclude the influence of other clinical and treatment related prognostic factors as compared to our quantitative PET metrics. In spite of these limitations, we present statistically significant data correlating quantitative interim-treatment PET metrics with clinical progression.

In conclusion, quantitative assessment of FDG-PET status after chemotherapy will likely be helpful for identifying patients at high risk of treatment failure at an early time point when treatment intensification could be considered. The preliminary findings of our study supports the quantitative interpretation of FDG-PET images as possibly an important tool guiding the design of prospective clinical trials of functional imaging for Hodgkin's disease.

## Competing interests

The authors declare that they have no competing interests.

## Authors' contributions

DT and LPR carried out the clinical review required in the study, analyzed the data and drafted the manuscript. ZS preformed the statistical analysis. RA, SH, RTH and AQ participated in the treatment of the patients included in the study. RA, RTH, AQ and EEG participated in the review of the drafted manuscript. BWL and PTT conceived of the study, participated in its design, performed the analysis and coordinated and helped to draft the manuscript. All authors read and approved the final manuscript.
